# Surveillance of strongyloidiasis in Spanish in-patients (1998–2014)

**DOI:** 10.1371/journal.pone.0189449

**Published:** 2017-12-28

**Authors:** Moncef Belhassen-García, Montserrat Alonso-Sardón, Angela Martinez-Perez, Cristina Soler, Cristina Carranza-Rodriguez, José Luis Pérez-Arellano, Antonio Muro, Fernando Salvador, on behalf of The Soil-Transmitted Helminths Study Group of the SEMTSI

**Affiliations:** 1 Servicio de Medicina Interna, Sección de Enfermedades Infecciosas, Complejo Asistencial Salamanca (CAUSA), Salamanca, Spain; 2 Centro de Investigación de Enfermedades Tropicales de la Universidad de Salamanca (CIETUS), Salamanca, Spain; 3 Institute for Biomedical Research of Salamanca (IBSAL), University Hospital of Salamanca, University of Salamanca, Spanish National Research Council (CSIC), Salamanca, Spain; 4 Area de Medicina Preventiva y Salud Pública, Facultad de Medicine, Universidad de Salamanca, Salamanca, Spain; 5 Consorci de Atenció Primaria de Salut Barcelona Esquerra, Hospital Clinic, Barcelona, Spain; 6 Unitat de Medicina Tropical i Salut Internacional, Servei de Medicina Interna, Hospital de Santa Caterina, Girona, Spain; 7 Unidad de Enfermedades Infecciosas, Complejo Hospitalario Universitario Insular Materno Infantil, Las Palmas de Gran Canaria, Spain; 8 Laboratorio de Inmunología Parasitaria y Molecular, Facultad de Farmacia, Universidad de Salamanca, Salamanca, Spain; 9 Infectious Diseases Department, Vall d'Hebron University Hospital, PROSICS, Barcelona, Spain; McGill University Health Centre, CANADA

## Abstract

**Background:**

*Strongyloides stercoralis* is a parasite that causes strongyloidiasis, a neglected tropical disease. *S*. *stercoralis* is a soil-transmitted helminth that is widely distributed in tropical and subtropical regions of the world. Strongyloidiasis can occur without any symptoms or as a chronic infection characterized by mild, unspecific symptoms such as pruritus, abdominal pain or discomfort; respiratory impairment also may manifest as a potentially fatal hyperinfection or disseminated infection. Most studies on strongyloidiasis in Spain have been related to chronic forms in immigrants or travellers from endemic zones and have mainly analysed out-patient populations. Studies of the impact of strongyloidiasis cases admitted to hospitals in Spain are lacking. Therefore, the aim of this study was to analyse the impact of strongyloidiasis in hospital care in Spain.

**Methodology:**

We designed a retrospective descriptive study using the Minimum Basic Data Set (MBDS, CMBD in Spanish) for inpatients with ICD-9: 127.2 (strongyloidiasis) diagnoses admitted to hospitals in the Spanish National Health System between 1998 and 2014.

**Principal findings:**

A total of 507 hospitalizations with diagnosis of strongyloidiasis were recorded, 324 cases (63.9%) were males. The mean (±SD) age was 42.1±20.1 years. The impact of strongyloidiasis on the total population of Spain was 0.06 cases per 10^5^ person-years, and the infection burden increased progressively over time (from 0.01 cases per 10^5^ person-years in 1999 to 0.10 cases per 10^5^ person-years in 2014). 40 cases (7.9%) died. The total cost was approximately €8,681,062.3, and the mean cost per patient was €17,122.4±97,968.8.

**Conclusions:**

Our data suggest that strongyloidiasis is frequent in Spain and is increasing in incidence. Therefore, it would be desirable to improve the oversight and surveillance of this condition. Due to the fact that strongyloidiasis can be fatal, we believe that there is a need to establish risk categories for inclusion in national guidelines/protocols for screening individuals at risk of developing strongyloidiasis.

## Introduction

Strongyloidiasis is mainly caused by *Strongyloides stercoralis*. Its transmission occurs in areas where poor hygienic conditions and humid, warm climate permit the free-living cycle of the parasite. Strongyloidiasis is usually acquired by walking barefoot on infested soil and is an endemic infection in the tropics and subtropics. The existing information suggests that *S*. *stercoralis* infections affect between 10% and 40% of the population in many tropical and subtropical countries. The global prevalence of *S*. *stercoralis* has increased within recent years due to infections in many endemic areas, specially Eastern, Southern and central Europe, Caribbean Islands, Southeast Asia, Latin America and sub-Saharan Africa[1,2]. The continued increase in the infection rate is solely attributed to poor personal hygiene, insufficient drinking water supply, unsatisfactory sanitary measures, and a lack of knowledge about the disease in high-risk populations[1]. In Europe, mainly in the Mediterranean area[3,4] including Spain, autochthonous cases have been reported[5,6], although most of the described cases are imported[7]. Strongyloidiasis can be suspected in symptomatic patients with digestive, respiratory or cutaneous complaints; however, asymptomatic eosinophilia and even ‘silent’ infections constitute the vast majority of infections in endemic areas[7]. Strongyloidiasis is one of the most difficult parasitic diseases to diagnose because suffer the lack of a diagnostic gold standard and relies on traditional diagnostic methods based on the visualization of *S*. *stercoralis* in stools (direct stool examination *vs* formalin-ethyl acetate concentration *vs* Harada mori, *vs* Baermann concentration *vs* agar plate culture) with different performance. Moreover, serologic tests have also different performance, recent tests developped with recombinant antigen demonstrate very good sensitivity and specificity. Also stool PCR is a promising diagnostic method. In addition, the sensitivity and specificity of this method vary among different patient groups[8]. In humans the larvae can also penetrate the skin or the intestinal mucosa to establish a cycle of repeated endogenous reinfection, this endogenous autoinfection cycle can possibly persist for a lifetime[9].The distinction between disseminated infection and hyperinfection is not strictly defined, but the hyperinfection syndrome implies the presence of signs and symptoms attributable to increased larval migration, with a increase larval burden but without the spread of the larvae outside the usual migration pathway, and disseminated disease implies larval migration to other organs. Development or exacerbation of gastrointestinal and pulmonary symptoms has been observed, and the detection of increased numbers of larvae in stool and sputum is the hallmark of hyperinfection. Hyperinfection syndrome has been described as late as 64 years after an individual has left an endemic area[9,10]. Both the hyperinfection and disseminated forms can result with fulminant and frequently fatal clinical presentations in immunosuppressed patients (hematological malignancies, transplant recipients, human T-cell lymphotropic virus type 1 (HTLV-1) infected patients, and those on corticosteroid treatment)[1,11].

In general, data on the impact of strongyloidiasis cases admitted to hospital are scarce[12–14]. The aim of this study was to analyse the impact of hospital cases of strongyloidiasis in Spain and to determine the evolution of strongyloidiasis incidence between 1998 and 2014.

## Materials and methods

The design was a retrospective descriptive study for inpatients classified under the diagnosis code ICD-9: 127.2 (strongyloidiasis) admitted to hospitals within the Spanish National Health System between 1998 and 2014. The National Health System (NHS) provides free medical care to 99.5% of the Spanish population, although those persons not covered by the NHS can receive care at public hospitals. Since 2005, the NHS surveillance and epidemiology department has provided patient data through the *Conjunto Minimo Basico de Datos* (CMBD as in Spanish), which is a national version of the European Minimum Basic Data Set (MBDS) that receives some information from private hospitals. It's the largest administrative database of inpatient and main source of information on morbidity, according to the demographic characteristics of the patients. MBDS containing valuable information on multiple aspects of hospital activity, as diagnoses, type of patients that cause longer stays and suppose a higher hospital cost, type of income (emergent or programmed), surgical procedures, type of financing and distribution of resources, quality and variability of care practice. In addition, it allows us to analyse the existing combinations between **main diagnosis** *(pathological process that is considered the main cause or reason for the patient's admission to the hospital*, *according to optional criteria)*, it's codified by the *"International Statistical Classification of Diseases—9 revision—clinical modification"* (ICD-9-MC), and **secondary diagnoses**, coexist with it at the time of admission, or develop throughout the hospital stay, and influence the duration of treatment or treatment), also they encoded by the ICD-9-MC. (Ministry of Health, Social Policy and Equality, http://www.mspsi.es/estadEstudios/estadisticas/cmbd/informes/notasMetodologicas.htm.)

This study analysed the data provided by one of existing surveillance systems in Spain, the MBDS. Therefore, we work with existing primary data records, we don’t generate the data. The MBDS includes data from all hospital discharges produced in the general hospital network of the NHS. The data contained in these records are those established in the above-mentioned MBDS and provide information on the characteristics of the patients treated (age and sex) and the appropriate variables as related to the hospitalization episode. Also, it collects the variable “average cost”, corresponds to the estimated average cost for each GRD in the process of estimating weights and costs of the NHS of the version in force, calculated or updated for the reference year. The MBDS is a universal and mandatory registry. Thus, the MBDS provides us information about 532 patients who have been admitted to the hospital during the years 1998–2014, and we collected information regarding hospital admissions with the strongyloidiasis code (ICD-9: 127.2) in diagnostic discharge reports. When patients were admitted with the same diagnosis several times, we registered only the first admission for analysis. Twenty-five patient records with missing data, such as age, gender or the city/region of residence, and patients with diagnosis mixed intestinal helminthiasis (ICD-9: 127.8) were **excluded** from the study. Finally, we **included** in this study 507 hospitalized patients in the Spanish National Health System between 1998 and 2014 with diagnoses of strongyloidiasis (ICD-9: 127.2). To improve the analysis, the patients were stratified according to gender and age (0–9, 10–19, 20–29, 30–39, 40–49, 50–59, 60–69, 70–79, 80–89, and 90–99 years). The data were analysed anonymously.

### Statistical analysis

The incidence rates were calculated by dividing the number of new cases of disease *(numerator)* per year/period by the population at risk *(denominator)* during a period of time (person-years) multiplied by 100,000 and expressed as “cases per 10^5^ person-years”. As it is not possible to accurately measure the disease-free periods, the total figure of the person-time at risk can be satisfactorily estimated when the size of the population is stable by multiplying the average population size studied by the duration of the observation period. Thus, the population at risk was obtained from annual data published by the National Institute of Statistics (INE, http://www.ine.es/) and the General Secretary of Immigration and Migration (Ministry of Employment and Social Security, http://extranjeros.empleo.gob.es/es/Estadisticas/index.html).

The incidence rates were computed by region and year in order to assess temporal and geographical patterns. The results in terms of mean rates by autonomous community were plotted in maps for the whole study period. The trends in hospitalization rates were analysed by linear regression for the whole study population and by age groups.

The results were expressed as a percentage for categorical variables and as the mean and standard deviation (SD) or median, 25^th^ and 75^th^ percentiles for continuous variables. A chi-squared test was used to compare the association between categorical variables, such as clinical and demographics variables, and the measured outcome was expressed as the odds ratio (OR) together with the 95% CI for OR. Continuous variables were compared with Student’s t-test or the Mann-Whitney test for two groups, depending on their normal or non-normal distributions. Additionally, we applied the corresponding Logistic Regression Model for multivariate analysis (dependent variable: qualitative or categorical), which allows us, from the *regression coefficients (β)* of the independent variables introduced in the model, to directly obtain the OR of each one of them. The construction of the logistic model was carried out by a *"forward"* type automatic procedure, based on the *likelihood ratio test*. The goodness check of the fit of the final model was based on the guidelines of Hosmer and Lemeshow. Thus, what is intended is to express the probability of occurrence of the event as a function of variables that are presumed relevant or influential. We considered a statistically significant difference from chance at a p-value <0.05. Data analysis was performed using SPSS 23 (*Statistical Package for the Social Sciences*).

### Ethics statement

This study involves the use of patient medical data from The MBDS. These data are hosted by the Ministry of Health Social Services and Equality (MSSSI). Researchers working in public and private institutions can request the databases by filling a questionnaire available on the MSSSI website. In this questionnaire, a signed Confidentiality Commitment is required. All of the data are anonymized and identified by the MSSSI before they are provided to applicants. According to this Confidentiality Commitment signed with the MSSSI, researchers cannot provide the data to other researchers; other investigators must request the data directly from the MSSSI[15].

The study protocol was approved by the Ethical Review Board of the Vall d’Hebron University Hospital (Barcelona, Spain). The procedures described here were performed in accordance with the ethical standards described in the Declaration of Helsinki as revised in 2013.

## Results

**Fig 1:**
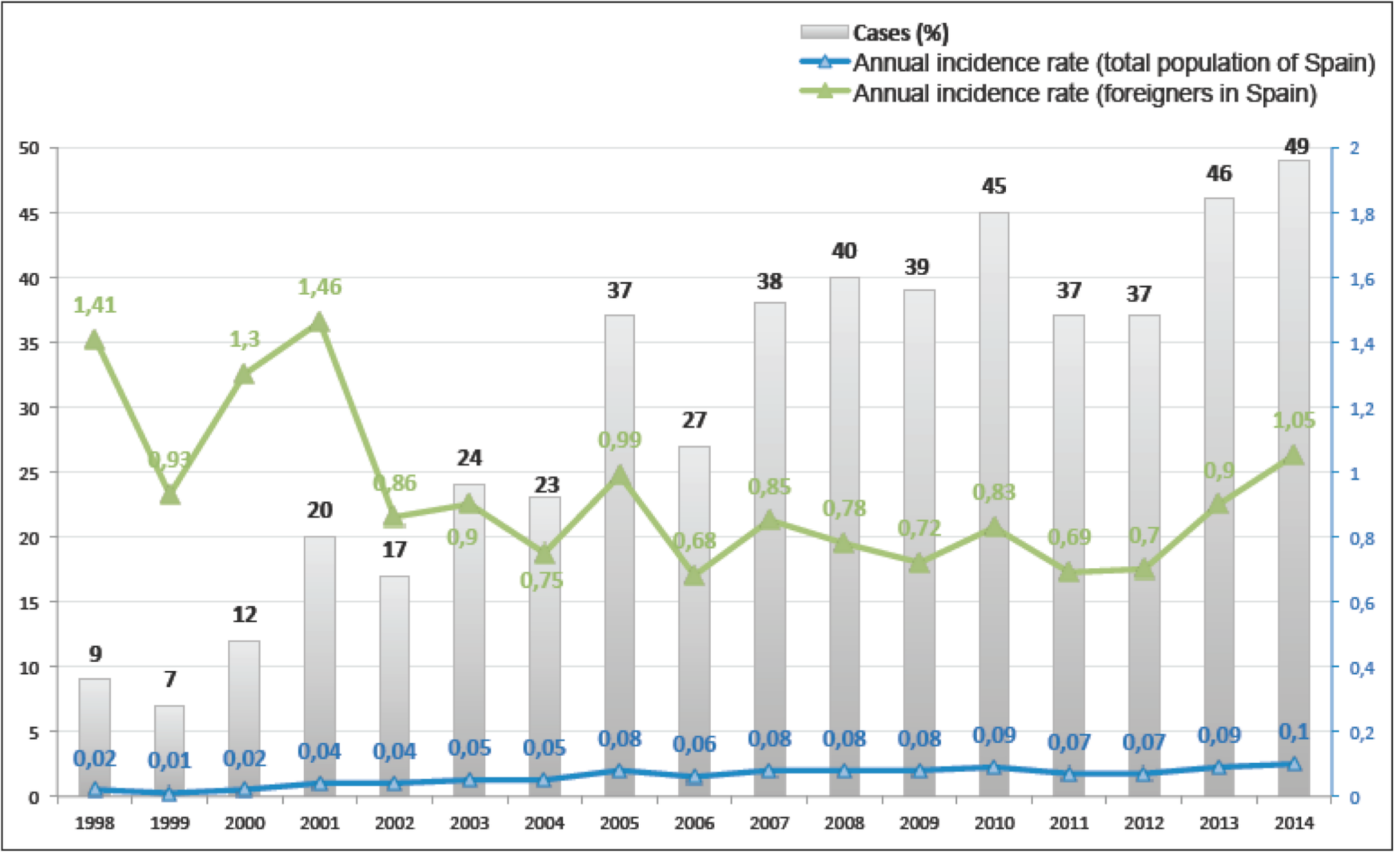
Temporal distribution of cohort during the study period: Cases and anual incidence rate *(cases per 105 person-years*). Data of Minimum Basic Data Set 1998–2014.

According to the inclusion criteria, 507 patients were hospitalized in the Spanish National Health System between 1998 and 2014 with diagnoses of strongyloidiasis (ICD-9: 127.2). **Fig 1** shows the annual distribution of cases during the study period and the annual incidence rates.

**Fig 2:**
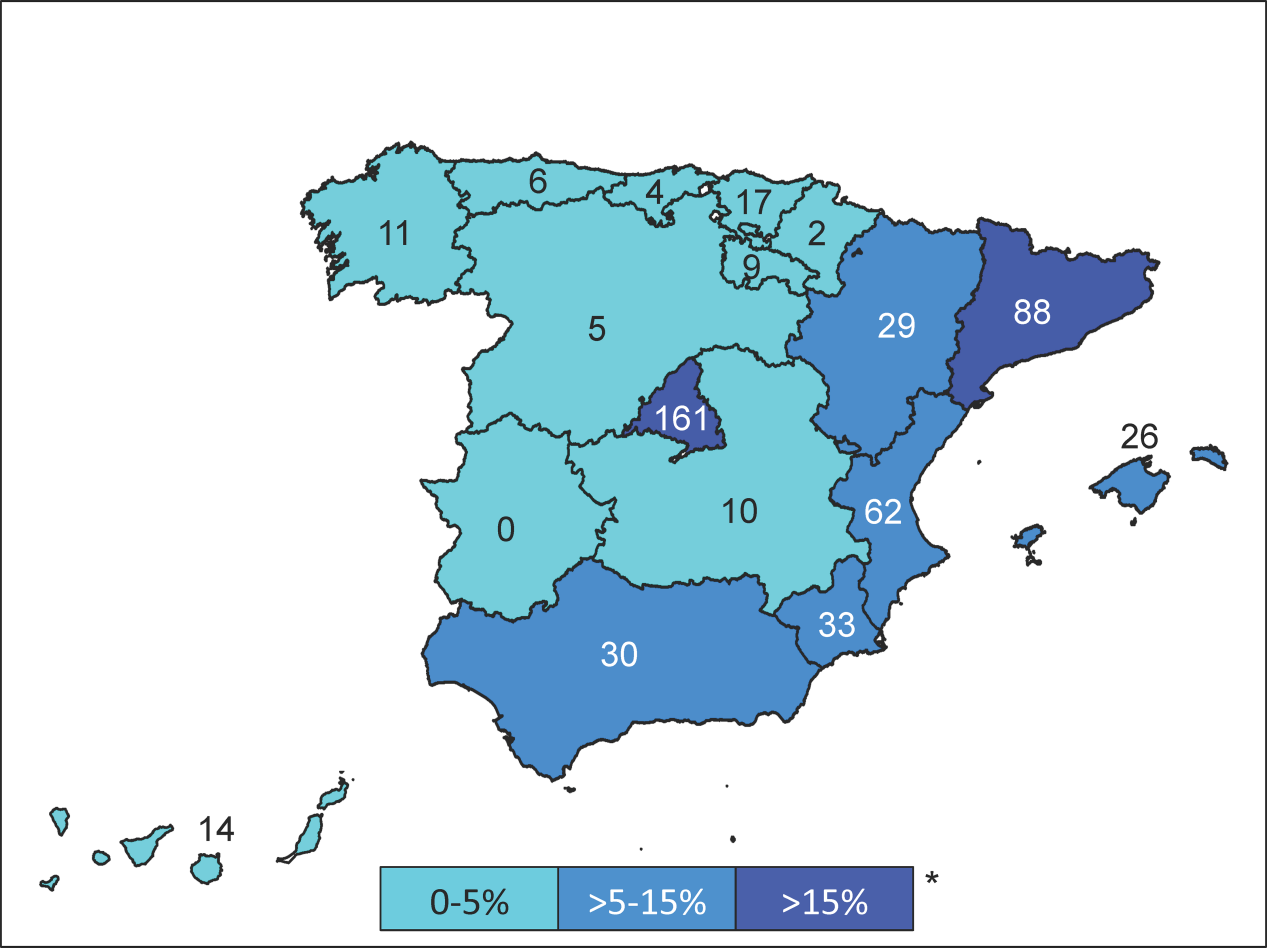
Number of cases and distribution of the cohort according to the autonomous community to which the hospital belongs 0–5%.

It shows that the number of cases increased over time (from 9 (1.8%) cases in 1998 to 49 (9.7%) cases in 2014) as did the incidence rate (from 0.01 cases per 10^5^ person-years in 1999 to 0.10 cases per 10^5^ person-years in 2014). The incidence rate of strongyloidiasis cases admitted to the hospital relative to the total population of Spain was 0.06 cases per 10^5^ person-years. No data regarding the nationalities of the admitted patients were available, However, if we assume that most patients with disseminated strongyloidiasis are foreign-born, and attribute all cases observed in our data to this population, then the period incidence rate among foreign-born people in Spain would be 0.85 cases per 10^5^ person-years and the annual incidence rates for foreigners show in Fig 1. **Fig 2** shows the distribution of the cohort according to the regions in which each hospital belongs.

We estimated the period incidence rates in each of the regions to assess the incidence rates of strongyloidiasi*s* cases admitted to the hospital located within each area. The highest rates were observed in La Rioja (0.18 cases per 10^5^ person-years), Madrid (0.16 cases per 10^5^ person-years) and Murcia (0.15 cases per 10^5^ person-years).

**Fig 3:**
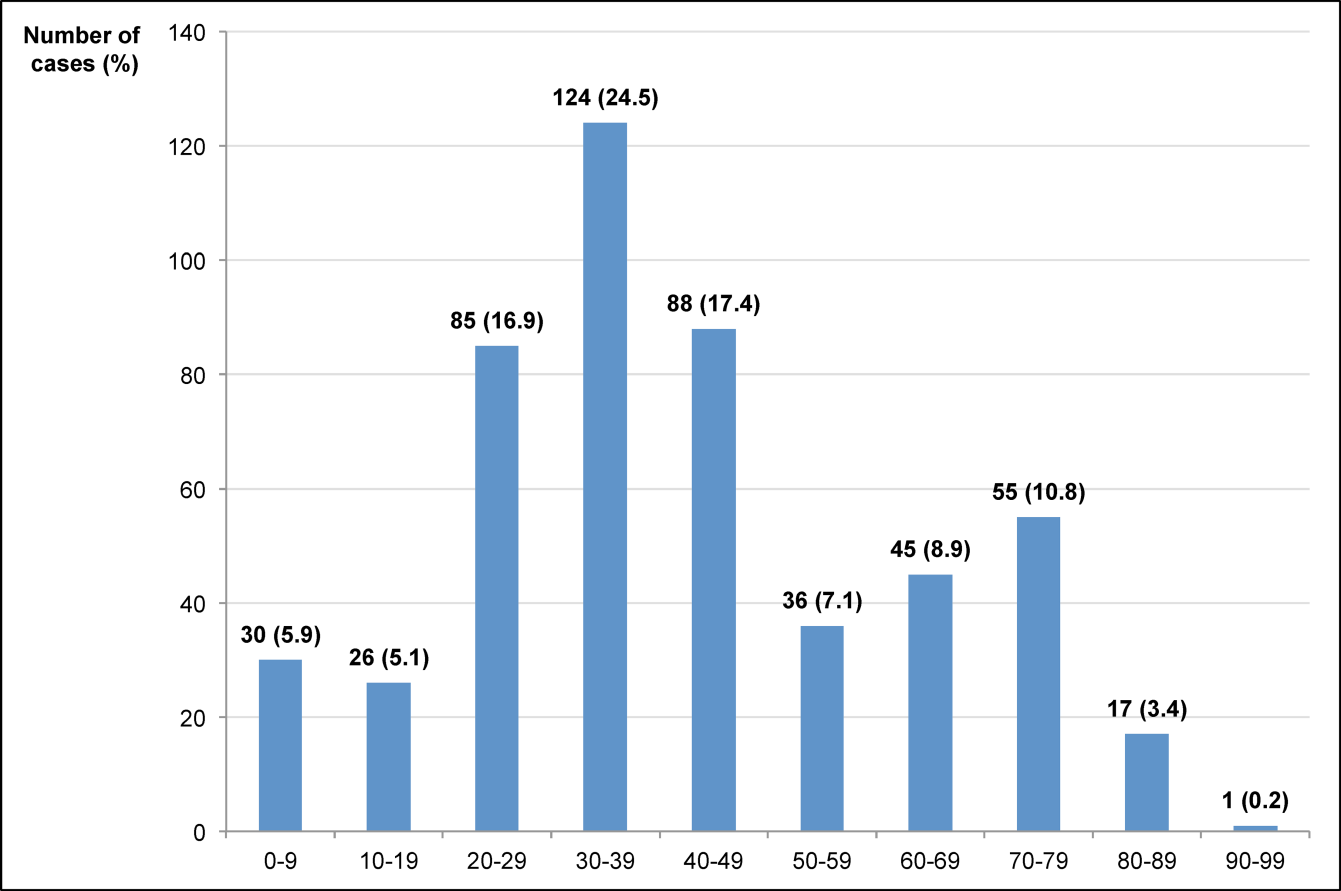
Distribution of the cohort by age groups.

Regarding gender, 324 cases (63.9%) were males and 183 cases (36.1%) were females. The male/female ratio was 1.8. The mean (±SD) age was 42.1±20.1 years (range 0–96), the median (25^th^, 75^th^ percentiles) value was 38 (29, 56) years. The highest number of cases was observed in the 30- to 39-year-old age group (124 cases; 24.5%) as shown in **Fig 3**.

The main data for patients included in the study are summarized in **Table 1**.

**Table 1:** Main data in patients included in the study.

	ICD-9: 127.2 = 507 cases, n (%)
**Gender: male**	324 (63.9)
**Age (years), Mean ± SD (range) Median (25**^**th**^**, 75**^**th**^ **percentiles)**	42.1 ± 20.1 (0–96)
	38 (29, 56)
**Type of admission**	
From the emergency service	407 (80.3)
Scheduled admission	96 (18.9)
Other/unknown	4 (0.8)
**Type of discharge**	
Home	448 (88.4)
Transfer to another Hospital	9 (1.8)
Voluntary discharge	1 (0.2)
Transfer to social-health center	6 (1.2)
Other/unknown	3 (0.6)
Exitus	40 (7.9)
**Hospital financing regime**	
Social Security	467 (92.1)
Private	29 (5.7)
Mutual Healthcare	1 (0.2)
Work accidents	1 (0.2)
Traffic accidents	1 (0.2)
Mixed financing	1 (0.2)
Other/unknown	7 (1.4)
**Hospital stay (days), Mean ± SD (range) Median (25**^**th**^**, 75**^**th**^ **percentiles)**	19.0 ± 26.0 (0–390)
	13 (7, 23)

In 407 cases (80.3%), hospital admission was an emergency, and in 96 cases (18.9%), the hospitalization was pre-planned. In relation to the services that provided inpatient hospital care, 198 cases (39.1%) were treated by *Internal Medicine*, 35 cases (6.9%) by *Infectious Diseases*, 20 cases (3.9%) by *Digestive Diseases*, 17 cases (3.4%) by *Paediatrics*, 16 cases (3.2%) by *Haematology* and 15 cases each (3.0%) by *Intensive*, *Nephrology* and *Pneumology*. The mean hospital stay (SD) was 19.0±26.0 days (range 1–390 days), the median (25^th^, 75^th^ percentiles) value was 13 (7, 23) days.

In 149 cases (29.4%), strongyloidiasis was the primary diagnosis/main diagnosis that caused hospitalization, in 115 (22.7%) cases, strongyloidiasis was a secondary diagnosis in 80 (15.8) strongyloidiasis was a third diagnosis, and in 163 (32.2%) cases was fourth diagnosis or superior.

Most patients (494, 97.4%) had comorbidity. We have collected information about the other diagnoses/comorbidities would have been relevant, as *Infectious and parasitic diseases* (ICD-9, codes 001–139) or *Malignant neoplasm of lymphatic and hematopoietic tissue* (ICD-9, codes 200–208) and we present in **Table 2**.

**Table 2:** Comorbidity associated with strongyloidiasis (127.2).

CODES	DISEASES	Freq.	%
(001–139) INFECTIOUS AND PARASITIC DISEASES			
001–009	Intestinal infectious diseases	53	10.4
010–018	Tuberculosis	45	8.9
020–027	Zoonotic bacterial diseases	2	0.4
030–041	Other bacterial diseases	**126**	**24.8**
042	Human immunodeficiency virus (HIV) infection	**102**	**20.1**
045–049	Poliomyelitis and other non-arthropod-borne viral diseases and prion diseases of central nervous system	20	3.9
050–059	Viral diseases accompanied by exanthem	13	2.5
060–066	Arthropod-borne viral diseases	1	0.2
070–079	Other diseases due to viruses and chlamydiae	52	10.2
080–088	Rickettsioses and other arthropod-borne diseases	43	8.5
090–099	Syphilis and other venereal diseases	18	3.5
110–118	Mycoses	53	10.4
120–129*	Helminthiases*	**92**	**18.1**
130–136	Other infectious and parasitic diseases	19	3.7
137–139	Late effects of infectious and parasitic diseases	1	0.2
(200–208) MALIGNANT NEOPLASM OF LYMPHATIC AND HEMATOPOIETIC TISSUE			
200	Lymphosarcoma and reticulosarcoma and other specified malignant tumors of lymphatic tissue	2	0.4
201	Hodgkin's disease	2	0.4
202	Other malignant neoplasms of lymphoid and histiocytic tissue	7	1.4
203	Multiple myeloma and immunoproliferative neoplasms	5	1.0
204	Lymphoid leukemia	11	2.2
205	Myeloid leukemia	2	0.4

*Except 127.2 Strongyloidiasis

In these patients, infectious comorbidity was more frequent. Highlight, 102 patients (20.1%) co-infected with HIV (ICD-9, code 042), 92 patients (18.1%) co-infected with other *Helminthiases* (ICD-9, codes 120–129, except code 127.2 *Strongyloidiasis*), and in the group of *Rickettsioses and other arthropod-borne diseases* (ICD-9, codes 080–088), there were 23 cases (4.5%) co-infected with *Malaria* (ICD-9, code 084.0). After reviewing 507 cases, only one case (0.2%) was co-infected with HTLV-I (ICD-9, code 079.51), and none HTLV-II (ICD-9, code 079.52), and 2 cases (0.4%) meningeal tuberculosis (ICD-9, code 013), without specifying more information.

On the other hand, *Malignant neoplasm of lymphatic and hematopoietic tissue* presented 29 cases (5.7%) in total. Of them, 6 cases (1.2%) were *Other malignant lymphomas*, *unspecified site*, *extranodal and solid organ sites* (ICD-9, 202.80) and 1 case (0.2%) was *Other malignant lymphomas*, *intra-abdominal lymph nodes* (ICD-9, 202.83).

We observed a crude mortality of 40 cases (7.9%). The mortality among those 149 patients presenting strongyloidiasis as a first diagnosis was 4.7% (7 cases), meaning that a primary diagnosis of strongyloidiasis was associated with 17.5% of all deaths.

The risk of mortality in the cohort was three times higher (OR = 2.7; 95% CI, 1.3–5.5; p = 0.004) when the diagnosis of strongyloidiasis was among the first 3^rd^ in order, or later (28 *vs*. 12 dead). No significant differences between mortality and gender were observed (p = 0.238), but there were statistically significant differences regarding age in which the risk of death doubled after age 40 (OR = 1.9; 95% CI, 1.1–3.7; p = 0.050; 25 *vs*. 15 dead). The mean (±SD) age (range) of the patients who died was 49.1±20.25 (7–83) years *vs*. 41.51±20.07 (0–96) years in the rest of the cohort (p = 0.022). The highest mortality occurred in patients co-infected with Human Immunodeficiency Virus (HIV), 14 dead; 35.0%, (OR = 2.3; 95% CI, 1.1–4.6; p = 0.014). The *multivariate logistic regression model* confirmed that age is a risk marker [Exp(B) = 2.0; 95%CI, 1.02–3.92; p = 0.042] that associated with late diagnosis (3^rd^ or more) [Exp(B) = 2.8; 95%CI, 1.39–5.69; p = 0.004], doubles and triples mortality risk in these patients.

In relation to the hospital finances, 92.1% (467 cases) belonged to hospitals in the public Social Security System (**Table 1**). The total cost was approximately €8.681.062, and the mean cost per patient was €17.122.4±97.968.8 (2 cases cost €0 to 1 case had a maximum cost of €1.543.880). The 149 patients who presented strongyloidiasis as first diagnosis represented a total cost for our NHS of 832.521,34€, with a mean cost of 5.587,39€ (SD 21.436,41€, range 1.063,31–259.040€).

## Discussion

We described a series of 507 cases of strongyloidiasis admitted to hospitals between 1998 and 2014; the incidence rate was 0.06 cases per 10^5^ person-years. This rate increased ten-fold during the study period. Most cases were male (63.9%), and the mean age was 42.1±20.1 years. The mean hospital stay was 19.0±26.0 days. In 264 cases (52.1%), strongyloidiasis was ranked as the primary or secondary diagnosis and thus was most likely the cause of admission. The total cost of the series was approximately €8.681.062, with a mean cost of 5.587,39€ (SD 21.436,41€). The crude mortality was 7.9%, but the mortality risk increased for those aged above 40, those presenting strongyloidiasis among their first three diagnoses and those co-infected with HIV.

Strongyloidiasis is an emerging global infection, and one of the most neglected helminth infections[1]. In contrast with other parasites, *S*. *stercoralis* has a very particular autoinfection life-cycle entirely within the human host that leads to chronic infections remaining undetected for decades. In addition, *S*. *stercoralis* is the only helminth that can lead to systemic infection with high parasite densities and severe to potentially fatal complications, especially in immunosuppressed hosts.

*S*. *stercoralis* is an endemic world-wide, heterogeneously distributed parasite with estimate prevalence up to 100 million infected people[1]. Many epidemiological aspects such as the real burden of the mortality and morbidity of *S*. *stercoralis* infection are poorly understood[16], and information on infection rates/prevalence of the parasite is scarce[17].

Therefore, current rates of infection remain underestimated in many countries due to the following reasons[2]: *i)* the optimal diagnostic strategy, both for epidemiological surveys and for individual diagnosis and screening, has yet to be defined and certainly deserves further research[18], *ii)* inadequate diagnostic methods with a low sensitivities are used (stool microscopy such as formalin-ether concentration), and therefore, better tools are needed for a more correct estimation of *S*. *stercoralis* prevalence, using at least one of the best available diagnostic methods in stools such as Baermann or Koga agar plate culture and adding an accurate serologic test when possible, iii) low parasitic load[2], iv) uncertain clinical symptoms[1,19,20] and v) a low index of suspicion among health care providers, especially in non-endemic countries[11,21]

Few European studies have provided data on the prevalence, diagnosis and treatment of infections with *S*. *stercoralis*. Most reports have been focused on immigrants and refugees from endemic countries, either through population or hospital-based studies, and considering all of the data available, this infection is clearly underestimated[19]. Only a small number of cases have considered whether infections were autochthonous or imported by travellers. Immigrants accounted for up to 90% of the diagnoses and, therefore, surveillance systems and diagnostic programmes should be focused on that population[7,21]. Thus, in the series reported by Ramirez Olivenza *et al*., there were no autochthonous or disseminated cases[7]. In our study due to the methodological limitations, it was not possible to assess the nationality of the patients, although given the current situation of strongyloidiasis infection in Spain, most of the cases were immigrants or travellers[17].

Despite the high impact of strongyloidiasis cases admitted to hospital, we found no epidemiological studies describing the effect of these cases. The most severe cases have been reported in western countries or in other affluent countries where the prevalence of the infection is low, and about half the cases are seen in migrants[22]. In most African, Asian, and Latin American countries, reports of severe and fatal strongyloidiasis are lacking or exceedingly rare, meaning that most cases may be missed[16]. Many isolated case reports on the emergence of the disease in different parts of the world that are non-endemic for this infection are being published. Most of these case studies refer to disseminated cases or cases with hyperinfection syndrome and belong to patients with immunosuppressive diseases, such as those receiving corticosteroid therapy, organ transplant recipients, and patients with haematological malignancies or other debilitating diseases[1]. In our work, we showed that during the study period, the incidence rate of strongyloidiasis cases admitted to hospitals in Spain was 0.06 cases per 10^5^ person-years, a significant figure considering that most cases can be silent or present with scarce symptoms, which is likely to lead to an underestimation of total real prevalence. Therefore, the real impact may be greater, and the current numbers may be the tip of the iceberg for strongyloidiasis in Spain. The increase in incidence rate of Strongyloidiasis over time may be due to several reasons: an increase awareness among physicians overtime and better diagnostic procedure; an increase number of travellers to endemic countries and immigration from them; finally, there are more immunocompromised patients.

Strongyloidiasis is typically asymptomatic in immunocompetent hosts, despite chronic infection. In hosts with defects in cell-mediated immunity, such as solid organ transplant recipients, strongyloidiasis can manifest as hyperinfection syndrome and/or disseminated disease with a risk of sepsis that reaches 50% secondary to a bacteraemia, pneumonia, meningitis, or peritonitis usually caused by an enterophatogens or polymicrobial agent gaining access to sites beyond the intestinal tract via disrupted mucosa or by larval migration itself[11]. The mortality rate of hyperinfection/disseminated strongyloidiasis is exceptionally high and ranges from 50 to 80%[1]. Early and active treatment may improve patient outcomes, but the mortality rate still ranges up to 30–71%[23]. If untreated, the mortality rate of disseminated disease approaches 100%[24]. In our work, however, the global mortality reached 7.9%.

Thus, in our work, only 30% of patients had strongyloidiasis as the primary diagnosis, so a better diffusion of the available information is badly needed, and collaboration among different specialists is desirable in order to provide common and adequate guidelines and protocols for screening for strongyloidiasis before the initiation of immunosuppressive therapy should be considered[23]. In addition, treatment of the chronic infection is mandatory to prevent life-threatening infection in at risk patients[1,22].

Despite these figures, strongyloidiasis is not rare and not always perceived as a relevant health problem not only by the general population but also by the medical community[19].

Otherwise, encoding errors may exist and cannot be amended as the data included in the MBDS are irreversible. The MBDS does not provide information about parasite diagnoses of strongyloidiasis, and this impairs the quality of data.

The main limitation of our work was the initial selection and detection bias. Despite their limitations, administrative databases (such as MBDS) have proven to be a valuable source of information. MBDS validity depends on the quality and completeness with which the hospital discharge report has been completed. Even if the MBDS provided information from a network of hospitals that covers more than 99% of the population living in Spain, it only considers the cases admitted to public hospital care; cases in private clinics and outpatients (mainly immigrants) were not included in this study. MBDS are data collected at hospital discharge. It is impossible to analyze causality or temporal sequence. Therefore, it is very difficult to analyze the complications. Therefore, we can assume that the actual incidence of strongyloidiasis is even higher than the incidence estimated in this study. As some authors indicate, the incidence rate is curiously low in areas where irregular sub-Saharan immigration is greater[25]. The ICM-9 only describes strongyloidiasis as a whole entity and does not consider the different clinical spectrum of the disease, and it is therefore impossible to determine how many cases were really sick with strongyloidiasis and how many presented with *S*. *stercoralis* infection at a point during hospital admission without it being a cause of disease. Unfortunately, no nationality data were available, and thus, we cannot confirm whether these cases were likely to be autochthonous or imported. As an advantage, ours is the first multicentre report on the surveillance of strongyloidiasis cases admitted to hospitals in Spain (1998–2014). In addition, the MBDS has been proven to be a trusted source for many epidemiological studies as it provides reliable information to support decision-making based on the ICD-9 codification carried out by medical doctors without being subject to the lack of diagnostic tools in outpatient care, leading to under-diagnosis or reporting deficiencies. It remains dependent only on the population’s health seeking behaviours or healthcare accessibility.

In summary, our data suggests that strongyloidiasis in Spain is frequent and is increasing in incidence. Therefore, it would be desirable to improve the oversight and surveillance of this disease. Because strongyloidiasis can be fatal in immunocompromised individuals, we believe that risk categories must be established for inclusion in national guidelines/protocols for screening individuals as candidates for immune-suppressant therapies and immigrants with eosinophilia.

Database available as a supplementary file.

## Supporting information

S1 Database (SAV)

## References

[1] Puthiyakunnon S, Boddu S, Li Y, Zhou X, Wang C, Li J, et al Strongyloidiasis—an insight into its global prevalence and management. Simon GL, editor. PLoS Neglected Tropical Diseases. Public Library of Science; 2014;8: e3018 doi: 10.1371/journal.pntd.0003018 25121962 10.1371/journal.pntd.0003018 PMC4133206

[2] Schär F, Trostdorf U, Giardina F, Khieu V, Muth S, Marti H, et al Strongyloides stercoralis: Distribution Global and Factors Risk. Brooker S, editor. PLoS Neglected Tropical Diseases. Public Library of Science; 2013;7: e2288 doi: 10.1371/journal.pntd.0002288 23875033 10.1371/journal.pntd.0002288 PMC3708837

[3] Sánchez PR, Guzman AP, Guillen SM, Adell RI, Estruch AM, Gonzalo IN, et al Endemic strongyloidiasis on the Spanish Mediterranean coast. QJM: monthly journal of the Association of Physicians. 2001;94: 357–363. 11435631 10.1093/qjmed/94.7.357

[4] Román-Sánchez P, Pastor-Guzmán A, Moreno-Guillén S, Igual-Adell R, Suñer-Generoso S, Tornero-Estébanez C. High prevalence of Strongyloides stercoralis among farm workers on the Mediterranean coast of Spain: analysis of the predictive factors of infection in developed countries. American Journal of Tropical Medicine and Hygiene. 2003;69: 336–340. 14628954

[5] Scaglia M, Brustia R, Gatti S, Bernuzzi AM, Strosselli M, Malfitano A, et al Autochthonous strongyloidiasis in Italy: an epidemiological and clinical review of 150 cases. Bull Soc Pathol Exot Filiales. 1984;77: 328–332. 6488423

[6] Magnaval JF, Mansuy JM, Villeneuve L, Cassaing S. A retrospective study of autochthonous strongyloïdiasis in Région Midi-Pyrénées (Southwestern France). European journal of epidemiology. 2000;16: 179–182. 10845269 10.1023/a:1007632028471

[7] Ramírez-Olivencia G, Espinosa MÁC, Martín AB, Núñez NI, Las Parras de ER, Núñez ML, et al Imported strongyloidiasis in Spain. Int J Infect Dis. Elsevier; 2014;18: 32–37. doi: 10.1016/j.ijid.2013.09.009 24211226 10.1016/j.ijid.2013.09.009

[8] Bisoffi Z, Buonfrate D, Sequi M, Mejia R, Cimino RO, Krolewiecki AJ, et al Diagnostic accuracy of five serologic tests for Strongyloides stercoralis infection. Siddiqui AA, editor. PLoS Neglected Tropical Diseases. Public Library of Science; 2014;8: e2640 doi: 10.1371/journal.pntd.0002640 24427320 10.1371/journal.pntd.0002640 PMC3890421

[9] Prendki V, Fenaux P, Durand R, Thellier M, Bouchaud O. Strongyloidiasis in man 75 years after initial exposure. Emerging Infectious Diseases. 2011;17: 931–932. doi: 10.3201/eid1705.100490 21529417 10.3201/eid1705.100490 PMC3321755

[10] Hauber HP, Galle J, Chiodini PL, Rupp J, Birke R, Vollmer E, et al Fatal outcome of a hyperinfection syndrome despite successful eradication of Strongyloides with subcutaneous ivermectin. Infection. 2005;33: 383–386. doi: 10.1007/s15010-005-5060-x 16258873 10.1007/s15010-005-5060-x PMC7102170

[11] Abanyie FA, Gray EB, Delli Carpini KW, Yanofsky A, McAuliffe I, Rana M, et al Donor-derived Strongyloides stercoralis infection in solid organ transplant recipients in the United States, 2009–2013. Am J Transplant. 2015;15: 1369–1375. doi: 10.1111/ajt.13137 25703251 10.1111/ajt.13137 PMC4747246

[12] González A, Gallo M, Valls ME, Muñoz J, Puyol L, Pinazo MJ, et al Clinical and epidemiological features of 33 imported Strongyloides stercoralis infections. Transactions of the Royal Society of Tropical Medicine and Hygiene. Oxford University Press; 2010;104: 613–616. doi: 10.1016/j.trstmh.2010.06.001 20637483 10.1016/j.trstmh.2010.06.001

[13] Fernández Rodríguez C, Enríquez-Matas A, Sanchéz Millán ML, Mielgo Ballesteros R, Jukic Beteta KD, Valdez Tejeda M, et al Strongyloides stercoralis infection: a series of cases diagnosed in an allergy department in Spain. J Investig Allergol Clin Immunol. 2012;22: 455–457. 23101199

[14] Martín Sánchez AM, Hernández García A, González Fernández M, Afonso Rodríguez O, Hernández Cabrera M, Pérez-Arellano JL. [Intestinal parasitosis in the asymptomatic Subsaharian immigrant population. Gran Canaria 2000]. Revista clínica española. 2004;204: 14–17. 14746755 10.1157/13056786

[15] Herrador Z, Siles-Lucas M, Aparicio P, López-Vélez R, Gherasim A, Gárate T, et al Cystic Echinococcosis Epidemiology in Spain Based on Hospitalization Records, 1997–2012. Torgerson PR, editor. PLoS Neglected Tropical Diseases. Public Library of Science; 2016;10: e0004942 doi: 10.1371/journal.pntd.0004942 27547975 10.1371/journal.pntd.0004942 PMC4993502

[16] Bisoffi Z, Buonfrate D, Montresor A, Requena-Mendez A, Muñoz J, Krolewiecki AJ, et al Strongyloides stercoralis: a plea for action. Lammie PJ, editor. PLoS Neglected Tropical Diseases. Public Library of Science; 2013;7: e2214 doi: 10.1371/journal.pntd.0002214 23675546 10.1371/journal.pntd.0002214 PMC3649953

[17] Martínez-Pérez Á, López-Vélez R. Is strongyloidiasis endemic in Spain? Taylan Ozkan A, editor. PLoS Neglected Tropical Diseases. Public Library of Science; 2015;9: e0003482 doi: 10.1371/journal.pntd.0003482 25654324 10.1371/journal.pntd.0003482 PMC4318575

[18] Requena-Mendez A, CHIODINI P, Bisoffi Z, Buonfrate D, Gotuzzo E, Muñoz J. The laboratory diagnosis and follow up of strongyloidiasis: a systematic review. Bottazzi ME, editor. PLoS Neglected Tropical Diseases. Public Library of Science; 2013;7: e2002 doi: 10.1371/journal.pntd.0002002 23350004 10.1371/journal.pntd.0002002 PMC3547839

[19] Buonfrate D, Baldissera M, Abrescia F, Bassetti M, Caramaschi G, Giobbia M, et al Epidemiology of Strongyloides stercoralis in northern Italy: results of a multicentre case-control study, February 2013 to July 2014. Euro surveillance: bulletin européen sur les maladies transmissibles = European communicable disease bulletin. 2016;21: 30310 doi: 10.2807/1560-7917.ES.2016.21.31.30310 27525375 10.2807/1560-7917.ES.2016.21.31.30310 PMC4998510

[20] Khieu V, Schär F, Forrer A, Hattendorf J, Marti H, Duong S, et al High prevalence and spatial distribution of Strongyloides stercoralis in rural Cambodia. Bisoffi Z, editor. PLoS Neglected Tropical Diseases. Public Library of Science; 2014;8: e2854 doi: 10.1371/journal.pntd.0002854 24921627 10.1371/journal.pntd.0002854 PMC4055527

[21] Valerio L, Roure S, Fernández-Rivas G, Basile L, Martínez-Cuevas O, Ballesteros Á-L, et al Strongyloides stercoralis, the hidden worm. Epidemiological and clinical characteristics of 70 cases diagnosed in the North Metropolitan Area of Barcelona, Spain, 2003–2012. Transactions of the Royal Society of Tropical Medicine and Hygiene. Oxford University Press; 2013;107: 465–470. doi: 10.1093/trstmh/trt053 23783760 10.1093/trstmh/trt053

[22] Buonfrate D, Requena-Mendez A, Angheben A, Muñoz J, Gobbi F, Van den Ende J, et al Severe strongyloidiasis: a systematic review of case reports. BMC Infectious Diseases. BioMed Central; 2013;13: 78 doi: 10.1186/1471-2334-13-78 23394259 10.1186/1471-2334-13-78 PMC3598958

[23] Lam CS, Tong MKH, Chan KM, Siu YP. Disseminated strongyloidiasis: a retrospective study of clinical course and outcome. European journal of clinical microbiology & infectious diseases: official publication of the European Society of Clinical Microbiology. 2006;25: 14–18. doi: 10.1007/s10096-005-0070-2 16418832 10.1007/s10096-005-0070-2

[24] Mejia R, Nutman TB. Screening, prevention, and treatment for hyperinfection syndrome and disseminated infections caused by Strongyloides stercoralis. Current Opinion in Infectious Diseases. 2012;25: 458–463. doi: 10.1097/QCO.0b013e3283551dbd 22691685 10.1097/QCO.0b013e3283551dbd PMC3430846

[25] Carranza Rodriguez C, Escamilla-González M, Fuentes-Corripio I, Perteguer-Prieto M-J, Gárate-Ormaechea T, Pérez-Arellano J-L. Helmintosis y eosinofilia en España (1990–2015). Enfermedades infecciosas y microbiologia clinica. 2016;: 1–17. doi: 10.1016/j.eimc.2015.11.019 26827134 10.1016/j.eimc.2015.11.019

